# Health-related productivity losses increase when the health condition is co-morbid with psychological distress: findings from a large cross-sectional sample of working Australians

**DOI:** 10.1186/1471-2458-11-417

**Published:** 2011-05-31

**Authors:** Libby Holden, Paul A Scuffham, Michael F Hilton, Robert S Ware, Nerina Vecchio, Harvey A Whiteford

**Affiliations:** 1 School of Medicine, Griffith University; University Drive Meadowbrook, Queensland 4131, Australia; 2 Queensland Centre for Mental Health research, Queensland Health; Level 3 Dawson house, The Park, Wacol, Queensland 4076, Australia; 3 University of Queensland, School of Population Health; Herston Road Herston, Queensland 4006, Australia

## Abstract

**Background:**

The health condition of workers is known to impact on productivity outcomes. The relationship between health and productivity is of increasing interest amid the need to increase productivity to meet global financial challenges. Prevalence of psychological distress is also of growing concern in Australia with a two-fold increase in the prevalence of psychological distress in Australia from 1997-2005.

**Methods:**

We used the cross-sectional data set from the Australian Work Outcomes Research Cost-benefit (WORC) study to explore the impacts of health conditions with and without co-morbid psychological distress, compared to those with neither condition, in a sample of approximately 78,000 working Australians. The World Health Organisation Health and Performance Questionnaire was used which provided data on demographic characteristics, health condition and working conditions. Data were analysed using negative binomial logistic regression and multinomial logistic regression models for absenteeism and presenteeism respectively.

**Results:**

For both absenteeism and presenteeism productivity measures there was a greater risk of productivity loss associated when health conditions were co-morbid with psychological distress. For some conditions this risk was much greater for those with co-morbid psychological distress compared to those without.

**Conclusions:**

Co-morbid psychological distress demonstrates an increased risk of productivity loss for a range of health conditions. These findings highlight the need for further research to determine whether co-morbid psychological distress potentially exacerbates lost productivity.

## Background

### Workers' health impact on absenteeism and presenteeism

Health related productivity loss is a concern to researchers, policy makers and industry. There is a growing body of evidence regarding the impact of health conditions on work performance [1-9]. It has been repeatedly found that health conditions increase work-related absences (absenteeism) and/or decrease productivity while at work (presenteeism), creating a substantial economic burden on industry [1-11]. Studies that have considered presenteeism have found it accounts for a greater proportion of the productivity loss than absenteeism [11,12]. For example the associated absenteeism by chronic condition ranged from 0.9 to 5.9 hours in a 4-week period, whereas on-the-job work impairment ranged from a 17.8% to 36.4% decrement in ability to function at work. The total cost of chronic conditions was estimated to be 10.7% of the total labour costs with 6.8% attributable to work impairment alone [11].

### Growing rates of co-morbidity

International research demonstrates a growing prevalence of co-morbidity, reporting that prevalence increases significantly with age [13-15]; and indicating that patients with co-morbidity in general practice represent the rule rather than the exception [15]. For example, Fortin et al. reported the prevalence of having 2 or more health conditions in the 18-to 44-year, 45-to 64-year, and 65-year and older age-groups was, respectively, 68%, 95%, and 99% among women and 72%, 89%, and 97% among men [13].

### Co-morbid psychological distress worsens health and productivity outcomes

There is now considerable evidence that psychological distress is often co-morbid with other health conditions and can worsen health outcomes [16-22]. High levels of psychological distress is a key indicator of mental health problems and is highly correlated with the presence of a diagnosable mental disorder, particularly depression [20]. Psychological distress, as used here, refers to subjective emotional distress [20] expressed by symptoms of depression, anxiety, acute grief reactions, and various other mental health conditions. Psychological distress is highly prevalent in the community, as are mental health disorders. The Australian Bureau of Statistics reported that in the 2004-05 snapshot of mental health and well-being in Australian 13% of adults reported experiencing high/very high levels of psychological distress in the previous four weeks [23]. This report does not account for prevalence in adolescents. There is limited data available on the impact of psychological distress on productivity; however, as psychological distress is a strong indicator for a diagnosable mental disorder [20,24], we draw on literature exploring the effects of psychological distress as well as depression on productivity. Psychological distress has a negative impact on working capacity [20]. Co-morbid depression is associated with significantly increased disability days, [18,25] reduced likelihood of working full-time and increased likelihood of reduced productivity [25].

### Gaps in literature demonstrate the need for further research on the impacts of co-morbid psychological distress on productivity decrements

There is strong evidence that a large number of adults with a disease have co-morbid psychological distress (PD) or depression. Few studies have considered the impact of disease co-morbid with PD impacting on productivity [25-27]. This study aims to address this gap in knowledge by exploring the impacts on absenteeism and presenteeism for a range of health conditions with and without co-morbid psychological distress, compared to those with neither health condition, in a large sample of working Australians.

## Methods

### Study design

The Australian Work Outcomes Research Cost-benefit (WORC) project obtained a large cross-sectional data set of approximately 78,000 working Australians. The WORC dataset was used in this study to explore associations between health and worker productivity, both with and without co-morbid psychological distress. The WORC study has been comprehensively discussed previously [28,29].

### Study sample

The WORC study recruited 58 large Australian-based companies located in both urban and rural Australia. Ten industry groups were included with the largest samples coming from health, education, government, and finance. Data was collected between October 2004 and December 2005.

### Study measures

The study involved the application of the validated World Health Organisation Health and Productivity Questionnaire (HPQ). The HPQ identifies 28 self-reported health conditions [30,31]. It probes self-reported absenteeism and presenteeism rates; and screens for psychological distress using the Kessler 6 (K6) [2,24]. Absenteeism was measured by the number of days and part days missed from work in the previous four weeks. Part days were treated as 0.5 day. All days and part days were summed to a total number of days absent. No categories or dichotomized variable was created. Presenteeism was measured using a self-rated score of overall performance in the past four weeks using a 0-10 scale (0 = worst possible performance, 10 = best possible performance). A series of memory priming and decomposition questions were asked which were applicable across occupations. Then a series of internal anchoring questions followed to enable the respondent to make comparisons with their own average performance and with the performance of their co-workers. Finally, an overall self-reported work performance (presenteeism) score was self-assessed using the same 0-10 scale. This was then categorized into low, average & high performance and used to measure presenteeism. Most people tend to score themselves highly, therefore the following categories were used: a low score is < 6, an average score is 6-9 and a high score is 10 [32].

For this study, self-reported health conditions were coded as 'yes' if respondents reported having the condition and either currently or previously having received professional treatment for that condition, and 'no' if they reported never having had the condition. Respondents were excluded if they reported having a condition but never received treatment as these respondents may have incorrectly self-diagnosed the health problem. An average of 0.05% were excluded per condition. Self-reported conditions included in this study were: arthritis, asthma, back/neck pain, cancers (excluding skin cancer), chronic obstructive pulmonary disease (COPD), cardiovascular disease (CVD), drug and alcohol problems, diabetes, fatigue, high blood pressure, high cholesterol, migraine, obesity (BMI score > 31, calculated from self-reported height and weight), workplace injury in past 12 months that required medical treatment, and psychological distress (being a K6 score of 13 and above). The optimal cut point on the K6 was identified by Kessler as 0-12 Vs 13 or more. This cut point has a total classification accuracy was 0.92 [33]. A score of 13 or more represents severe psychological distress and indicates the likely presence of a mental health disorder. This cut point was used to create a dichotomous variable to indicate the presence or absence of high psychological distress where scores are ≥13 or 0-12 respectively. A person was coded as having co-morbid psychological distress if they had a K6 score of 13 or more and had one of the health conditions listed above. The person could have more than one health condition from the above list; this is adjusted for in the multivariate model using the number of co-morbidities covariate as listed below.

The covariates adjusted for in all models were: demographic characteristics of: age, sex, marital status, number of children, education level and annual income; health characteristics of: general treatment seeking behaviour (number of occasions of treatment for any reason except pregnancy, not treatment for a specific condition) and the number of co-morbid conditions; and working condition factors of: occupation, industry, public/private sector, job security (using proxy of part-time or full-time compared to casual), contractor, the rate of workplace accidents per 100 workers in the previous four weeks, hours worked in previous week, supervisory role (number of staff supervised), and hours expected to work in a 7 day week by their employer (as perceived by respondent).

### Statistical analysis

For each health condition, two regression models were developed, one for absenteeism, the other for presenteeism. To account for multiple co-morbidities, models adjusted for the number of co-morbid conditions using the groupings recommended by Kessler (no health conditions, one condition, two-four, five-seven, eight-ten or eleven or more conditions) [32].

For self-reported absenteeism a negative binomial logistic regression statistical procedure was used because it was the most suitable statistical test to cater for a continuous variable with inflated number of zero responses. Presenteeism was modelled using multinomial logistic regressions. The reference category was moderate work performance. High work performance results are not reported here as we are only interested in health associations with under-performance.

To explore the relationship of co-morbid psychological distress with the indexed health condition the variable is separated into three categories as follows: the reference category has neither the indexed health condition nor co-morbid psychological distress (but could also have other health conditions); the second category has the indexed health condition but not co-morbid psychological distress (but could have also other health conditions); and the third category has the indexed health condition and co-morbid psychological distress (and could also have other health conditions).

## Results

The response rate was 24.7% providing 90,279 responses. From this, a sample of 78,430 workers had complete absenteeism data; and a sample of 77,455 workers had complete presenteeism data. Respondents' demographic characteristics are described in Table 1. The sample included part-time, full-time and casual workers. Approximately 80% were aged 30-59 years. Sixty five percent were female, which is greater than the proportion in the Australian workforce [34]. The sample also has greater representation of workers from industries of health, education, and government administration; and fewer from retail, construction and mining [34]. The average income and education level are fairly representative of the Australian population [34].

**Table 1:** Demographic characteristics: 78,430 working Australians

Demographic Variables	%
AGE ^¥^	
18-29 years	17
30-44 years	43
45-59 years	37
60-70 years	3
SEX	
Female	65
Male	35
MARITAL STATUS	
Separated, divorced, widowed, never married	29
Married or cohabitating	71
NUMBER OF CHILDREN	
Nil	69
1-3 children	28
4 or more children	3
EDUCATION LEVEL	
Did not complete high school	14
Completed high school	10
Some college	27
Completed college or university	48
ANNUAL WAGE β	
≤$29999 pa	13
$30000-39999 pa	14
$40000-49999 pa	21
$50000-74999 pa	36
$75000-99999 pa	10
≥$100000 pa	7

¥: only persons aged 18-70 included in analysis; β: excludes hourly rate

< $7.50ph in case fortnightly income reported instead of annual income

### Absenteeism

Compared to the reference category (having neither the indexed condition nor co-morbid psychological distress (PD)), those with the indexed condition had a significantly increased incidence rate ratio (IRR) of absenteeism in unadjusted models (see Table 2). This was the case for all explored health conditions. For example: a person with arthritis but no psychological distress has a 40% higher risk of absenteeism compared to a person with neither arthritis nor psychological distress. However a person with both arthritis and psychological distress has a 124% higher risk of absenteeism".

**Table 2:** Unadjusted risk of Absenteeism for those with and without co-morbid psychological distress compared to the reference group that had neither the indexed health condition nor co-morbid psychological distress, using negative binomial logistic regression; reporting Incidence Rate Ratios (IRR)

		Without co-morbid PD		With co-morbid PD	
HEALTH CONDITION	Model n	%	IRR (95%CIs)	%	IRR (95%CIs)
Arthritis	64888	3.5	1.40** (1.32-1.49)	0.2	2.24** (1.73-2.92)
Asthma	65788	6.3	1.22** (1.17-1.28)	0.3	1.75** (1.43-2.14)
Back/neck pain	70579	29.6	1.22** (1.19-1.25)	1.5	2.02** (1.85-2.21)
Cancers	74514	3.1	1.27** (1.20-1.35)	0.14	2.36** (1.81-3.08)
COPD	71750	0.4	1.51** (1.27-1.79)	0.03	2.80* (1.55-4.92)
CVD	74002	0.8	1.17* (1.03-1.32)	0.06	2.14* (1.39-3.29)
Drug & Alcohol	72227	3.0	1.74** (1.30-2.32)	0.5	2.24** (1.47-3.44)
Diabetes	73853	2.0	1.18** (1.10-1.27)	0.12	1.60* (1.18-2.16)
Fatigue/sleep problems	74630	0.6	1.48** (1.29-1.69)	0.10	2.08** (1.51-2.87)
High Blood Pressure	71009	8.1	1.14** (1.10-1.19)	0.3	1.82** (1.52-2.18)
High Cholesterol	68637	5.7	1.10** (1.05-1.16)	0.2	1.87** (1.50-2.32)
Migraine/severe headache	70881	9.1	1.22** (1.17-1.26)	0.7	1.94** (1.71-2.20)
Obesity	71849	11.0	1.22** (1.18-1.27)	0.7	2.04** (1.79-2.32)
Workplace Injury	75031	7.0	1.43** (1.37-1.49)	0.5	2.66** (2.29-3.09)

*: p < 0.05; **: p < 0.001; † trend < 0.1

Those with the indexed condition and co-morbid PD also had a greater IRR than the reference category for all conditions explored. The IRR for those with co-morbid PD was greater than that of persons with the indexed condition and no co-morbid PD for all conditions explored. Conditions with the highest ranking IRR when not co-morbid with PD were drug and alcohol (IRR: 1.74: CI: 1.30-2.32), COPD, fatigue, injury, arthritis, and cancers. When co-morbid with PD, conditions with the highest ranking IRR were COPD (IRR: 2.80 CI: 1.55-4.92), injury, cancer, D&A, and arthritis.

The IRR effect sizes were reduced in adjusted models (see Table 3). Some health conditions, with or without co-morbid PD, no longer had a significantly increased IRR compared to the reference category (having neither the indexed condition nor co-morbid PD). These were asthma, COPD, CVD, and fatigue. Some conditions only demonstrated a significantly increased IRR when co-morbid with PD. These were back/neck pain and migraine. Conditions with the highest IRR effect without co-morbid PD were D&A (IRR: 1.32 CI: 1.00-1.75), injury, and arthritis. When co-morbid with PD the highest ranking conditions were cancers (IRR: 1.83 CI: 1.44-2.43), injury, and arthritis.

**Table 3:** Adjusted risk of Absenteeism for those with and without co-morbid psychological distress compared to the reference group that had neither the indexed health condition nor co-morbid psychological distress, using negative binomial logistic regression; reporting Incidence Rate Ratios (IRR)

		Without co-morbid PD		With co-morbid PD	
HEALTH CONDITION	Model n	%	IRR (95%CIs)	%	IRR (95%CIs)
Arthritis	62295	3.5	1.07* (1.01-1.13)	0.2	1.41* (1.08-1.83)
Asthma	63021	6.3	1.03 (0.99-1.08)	0.3	1.15 (0.94-1.40)
Back/neck pain	67715	29.6	1.02 (0.99-1.05)	1.5	1.33** (1.22-1.46)
Cancers	71446	3.1	1.08* (1.02-1.15)	0.14	1.83** (1.44-2.43)
COPD	68787	0.4	1.44 (0.96-1.35)	0.03	1.63 (0.91-2.91)
CVD	70954	0.8	0.96 (0.85-1.08)	0.06	1.34 (0.87-2.06)
Drug & Alcohol	69237	3.0	1.32* (1.00-1.75)	0.5	1.48^† ^(0.99-2.23)
Diabetes	70816	2.0	0.89*(0.83-0.96)	0.12	1.15 (0.85-1.55)
Fatigue/sleep problems	71555	0.6	0.99 (0.86-1.13)	0.10	1.28 (0.93-1.74)
High Blood Pressure	68091	8.1	0.94* (0.90-0.98)	0.3	1.19^† ^(1.00-1.42)
High Cholesterol	65817	5.7	0.93* (0.89-0.98)	0.2	1.27*(1.03-1.57)
Migraine/severe headache	67969	9.1	1.01 (0.97-1.05)	0.7	1.29** (1.14-1.46)
Obesity	68980	11.0	1.06* (1.02-1.09)	0.7	1.33** (1.17-1.51)
Workplace Injury	72095	7.0	1.19** (1.14-1.24)	0.5	1.46** (1.26-1.69)

Adjusting for: treatment-seeking behaviour, number of co-morbidities, income, industry, public/private sector, occupation, contractor, supervisory role, rate of work accidents per employer, age, education, sex, job security, hours worked in last week, marital status, children, and hours expected to work p.w. (as perceived by employee)

*: p < 0.05; **: p < 0.001; † trend < 0.1

### Presenteeism

In unadjusted models (see Table 4) all health conditions, when co-morbid with PD, had a significantly increased risk compared to the reference category (having neither the indexed condition nor co-morbid PD). Some conditions only had a significantly increased effect size when co-morbid with PD. These conditions were arthritis, cancers, CVD, high blood pressure, and high cholesterol. Conditions with the highest IRR when not co-morbid with PD were D&A (IRR: 2.57 CI: 2.25-2.94), fatigue, COPD, obesity and injury. Conditions with greatest effect sizes when co-morbid with PD were injury (IRR: 9.46 CI: 7.47-11.99), fatigue, D&A, arthritis, back/neck pain, diabetes, obesity, and COPD.

**Table 4:** Unadjusted Relative Risk Ratio (RRR) of Presenteeism for those with and without co-morbid psychological distress compared to the reference group that had neither the indexed health condition nor co-morbid psychological distress

		Without co-morbid PD		With co-morbid PD	
HEALTH CONDITION	Model n	%	RRR (95%CIs)	%	RRR (95%CIs)
Arthritis	64079	3.5	1.10 (0.90-1.33)	0.2	7.90** (5.21-11.97)
Asthma	64970	6.3	1.22* (1.06-1.40)	0.3	5.91** (4.20-8.30)
Back/neck pain	69701	29.6	1.22** (1.13-1.31)	1.5	7.83** (6.76-9.08)
Cancers	73585	3.1	0.87 (0.70-1.08)	0.14	7.36** (4.78-11.34)
COPD	70852	0.4	2.14** (1.41-3.24)	0.03	7.53** (2.92-19.43)
CVD	73083	0.8	1.47 (1.04-2.07)	0.06	7.10** (3.54-14.22)
Drug & Alcohol	74439	3.0	2.57** (2.25-2.94)	0.5	8.59** (6.81-10.84)
Diabetes	72935	2.0	1.33* (1.06-1.65)	0.12	7.60** (4.72-12.22)
Fatigue/sleep problems	73698	0.6	2.56** (1.89-3.46)	0.10	8.80** (5.35-14.46)
High Blood Pressure	70119	8.1	0.90 (0.79-1.04)	0.3	6.43** (4.75-8.69)
High Cholesterol	67801	5.7	1.02 (0.87-1.19)	0.2	7.31** (5.13-10.41)
Migraine/severe headache	69999	9.1	1.30** (1.16-1.45)	0.7	7.27** (5.94-8.90)
Obesity	70967	11.0	1.60** (1.42-1.73)	0.7	7.68** (6.22-9.48)
Workplace Injury	74086	7.0	1.35** (1.20-1.53)	0.5	9.46** (7.47-11.99)

Using multinomial logistic regression, reporting relative risk ratios for low productivity compared to average productivity (high productivity not reported)

*: p < 0.05; **: p < 0.001; † trend < 0.1

All conditions in adjusted models (see Table 5) were associated with an increased IRR when co-morbid with PD, compared to the reference category (having neither the indexed condition nor co-morbid PD). Some were only significant when co-morbid with PD. These were arthritis, asthma, cancers, diabetes, high cholesterol and migraine. The largest effect sizes for conditions when not co-morbid with PD were for D&A (IRR: 2.04 CI: 1.16-3.59), fatigue, and obesity. The largest effect sizes compared to the reference category for conditions that were co-morbid with PD were for: arthritis (IRR: 5.06 CI: 3.18-8.05), injury, cancers, and back/neck pain.

**Table 5:** Adjusted Relative Risk Ratio (RRR) of Presenteeism for those with and without

		Without co-morbid PD		With co-morbid PD	
HEALTH CONDITION	Model n	%	RRR (95%CIs)	%	RRR (95%CIs)
Arthritis	61081	3.5	0.90 (0.73-1.12)	0.2	5.06** (3.18-8.05)
Asthma	61785	6.3	0.88 (0.76-1.02)	0.3	2.59** (1.79-3.75)
Back/neck pain	66278	29.6	0.91* (0.83-0.99)	1.5	4.20** (3.53-4.99)
Cancers	70037	3.1	0.80^† ^(0.64-1.00)	0.14	4.44** (2.75-7.18)
COPD	67437	0.4	1.43 (0.92-2.23)	0.03	3.52* (1.30-9.54)
CVD	69564	0.8	1.17 (0.82-1.69)	0.06	3.58** (1.69-7.58)
Drug & Alcohol	67737	3.0	2.04* (1.16-3.59)	0.5	3.23** (1.64-6.36)
Diabetes	69422	2.0	0.96 (0.77-1.22)	0.12	3.62** (2.15-6.10)
Fatigue/sleep problems	70146	0.6	1.47* (1.06-2.04)	0.10	3.82** (2.23-6.56)
High Blood Pressure	66741	8.1	0.81* (0.69-0.93)	0.3	3.65** (2.63-5.07)
High Cholesterol	64532	5.7	0.87 (0.73-1.03)	0.2	4.03** (2.74-5.93)
Migraine/severe headache	66632	9.1	0.90^† ^(0.79-1.02)	0.7	3.57** (2.83-4.49)
Obesity	67640	11.0	1.24** (1.11-1.38)	0.7	3.90** (3.08-4.93)
Workplace Injury	70682	7.0	1.11^† ^(0.98-1.27)	0.5	4.83** (3.73-6.25)

co-morbid psychological distress compared to the reference group that had neither the indexed health condition nor co-morbid psychological distress. Using multinomial logistic regression, reporting relative risk ratios for low productivity compared to average productivity (high productivity not reported)

Adjusting for: number of co-morbidities, treatment-seeking behaviour, age, sex, education, income, marital status, children, income, occupation, industry, public/private sector, job security, contractor, hours worked in last week, supervisory role, rate of work accidents per employer, hours expected to work p.w. (as perceived by employee)

*: p < 0.05; **: p < 0.001; † trend < 0.1

## Discussion

Both individual health conditions and conditions when co-morbid with psychological distress had a greater impact on presenteeism than absenteeism in terms of the effect sizes in adjusted models. All health conditions had a greater risk of either absenteeism or presenteeism when co-morbid with psychological distress. The increase in the size of the risk when co-morbid with psychological distress was greater for presenteeism than absenteeism. A larger number of health conditions were associated with significant increased risk of absenteeism when not co-morbid with psychological distress compared to presenteeism (7 and 3 conditions respectively). However, when co-morbid with psychological distress all 14 health conditions were significantly associated with productivity loss for presenteeism compared to 7 of 14 for absenteeism.

These findings suggest a greater than additive effect for some health conditions, particularly those that demonstrate a two-five fold increased IRR. Although the literature on psychological distress is relatively sparse, there is a growing body on depression. Note that the measure of psychological distress is a composite of anxiety, depression, acute grief reactions and various other mental health conditions, and therefore, we are able to link our outcomes with studies that include questionnaires for depression. The literature supports the finding that co-morbid depression can have an additive effect [18,25] and a greater than additive effect [26,35,36]. Co-morbid physical-mental health problems has reportedly led to a mainly additive increase in work-loss [18,25], reduced likelihood of full-time working status [25], and a significant increase in disability days [25].

The current study found that cancers when co-morbid with psychological distress had a significant impact on productivity losses for both absenteeism and presenteeism; no previous studies have been found that explore the impacts of co-morbid cancer and psychological distress on productivity. Arthritis and workplace injury were both found in our study to be associated with an increased risk of both absenteeism and presenteeism when co-morbid with psychological distress. A study exploring associations with depression across a number of health conditions and their impacts on productivity losses [26] found no significant impact on role impairment when depression is co-morbid with injuries; however, this study found a significant impact on both productivity measures.

Several studies report that co-morbid depression and COPD have a significant impact on productivity loss [25,37]. However, in this study the impact was only significant for presenteeism. A review of the link between depressive disorders and health condition reported that obese women had a 50% increased lifetime prevalence of depressive disorders [22]. No studies were found that explored the impact of obesity co-morbid with psychological distress on productivity. However one study that focused specifically on productivity losses related to obesity adjusted for the number of co-morbid conditions [38]. This indicates the importance of co-morbidity on lost productivity estimates for obesity. The current study found obesity when co-morbid with PD to be significantly associated with both increased presenteeism and absenteeism.

Co-morbid mental disorders and substance use disorders are prevalent in 12% of people presenting to general practice in Australia [39,40]. Those with this co-morbidity were found to have more days out of role than people with either of these conditions in isolation [40] but little is known about the impact of this co-morbidity on productivity. This study found a significant increased Relative Risk Ratio (RRR) of presenteeism when D&A is co-morbid with PD and a trend of increased IRR for absenteeism (p = 0.06).

Co-morbid depression and CVD has been reported to impact on work absence [37] and on role impairment [25]. This study found the impact of psychological distress and CVD was significant for presenteeism only. Consistent with our findings, fatigue has been found to impact on work performance when co-morbid with depression [30]. Depression has been found to have a potential mediating effect on the relationship between fatigue and absenteeism for persons with insomnia [41]. Other studies have adjusted for co-morbid depression when exploring the impacts of fatigue on productivity losses [42-44] demonstrating the importance of co-morbid depression on productivity losses for this condition. This study found fatigue, when co-morbid with PD significantly impacted on presenteeism but not absenteeism.

Some conditions were found to demonstrate a protective effect when considered independently, but many demonstrated an increased likelihood of productivity decrement when co-morbid with psychological distress. These were high blood pressure, and high cholesterol for absenteeism; and back/neck pain and high blood pressure for presenteeism. Diabetes also demonstrated the same pattern but not at a statistically significant effect size. These findings are consistent with the literature in that, employees with depressive illness and either heart disease, diabetes, hypertension or back problems were found to cost the employer 1.7 times more than those with the comparative condition alone [27]. One study found that employees with diabetes had a 2.15-fold excess risk of absenteeism but that up to 55% was attributable to depression and only 7% was purely attributable to diabetes [35].

There are some limitations to our study that need to be considered. Associations between factors were determined in this cross-sectional study; however, no conclusions can be drawn regarding causal pathways. The sample has more white-collar workers than the general population. Industry type and work role were included in models to adjust for these potential differences; however, extrapolation of these findings to the general employed population should be undertaken only where there is a clear match in the demographics of any sub-group. It should also be considered that only those at work during the data collection period responded. It could be that people on extended sick leave or out of the workforce are not represented. This may explain our cancer results, as other studies have demonstrated a strong association between productivity losses and cancer [1]. Other limitations include the self-reported nature of health conditions, the over-representation of females, the absence of some top burden of disease conditions such as kidney diseases, and the absence of some relevant work-related characteristics such as decision-making control. In addition, the survey was conducted from October 2004 to December 2005 and included one summer holiday season over the Christmas and New Year period in Australia. We were unable to control for any potential seasonal effects as many participants did not include the date the survey was completed. Nevertheless, many parts of Australia are tropical and sub-tropical, so the seasonal effects on illness are less pronounced compared with temperate climates. Strengths of the study include the large sample size, the range of data available relating to health conditions, work-related characteristics, demographic characteristics and the sample representing regional, rural and urban Australia.

This research raises the question of whether psychological distress is a potential mediating factor in lost productivity. Although with cross-sectional data we cannot conclusively answer this question; our findings highlight to need for further research into whether psychological distress, a treatable condition, may well exacerbate productivity losses. There is now strong evidence for the association between mental health problems, particularly depression, and other health conditions [16,21,45]; and between depression and productivity loss [5,46-48].

## Conclusions

This research suggests that psychological distress is an exacerbating factor in lost productivity. Given the evidence for a growing prevalence of psychological distress [49], and the role this plays in mediating sickness absence for other diseases [50], including chronic back pain and arthritis [51], this area is an important one for further research with appropriate study designs, to enable the development of interventions that can be applied to the wider community.

## List of abbreviations

CI: Confidence intervals; COPD: Chronic obstructive pulmonary disease; CVD: Cardio-vascular disease; D&A: Drug and alcohol problems; HPQ: World Health Organisation Health and Productivity Questionnaire; IRR: Incidence rate ratio; K6: Kessler 6 (Psychological Distress questionnaire); PD: Psychological distress; RRR: relative risk ratio; WORC: Australian Work Outcomes Research Cost-benefits (WORC) study

## Competing interests

The authors declare that they have no competing interests.

## Authors' contributions

*LH *developed the methods for this study in collaboration with co-investigators, conducted data analysis with help from statistician, wrote first draft and revisions to paper, corresponding author; *PS *assisted with developing methods, advised on statistical analysis methods, and reviewed drafts of paper; *MH *coordinated data collection and reviewed drafts of paper. *RW *assisted with data analysis and reviewed drafts of paper; *NV *assisted with developing methods for this study and reviewed drafts of paper; and *HW *chief investigator of parent study which collected the data used in this study and reviewed drafts of paper. All co-authors read and approved the final manuscript.

## Pre-publication history

The pre-publication history for this paper can be accessed here:

http://www.biomedcentral.com/1471-2458/11/417/prepub
